# Differential secretome analysis of *Pseudomonas syringae pv tomato* using gel-free MS proteomics

**DOI:** 10.3389/fpls.2014.00242

**Published:** 2014-07-04

**Authors:** Jörg Schumacher, Christopher J. Waite, Mark H. Bennett, Marcos F. Perez, Kishwar Shethi, Martin Buck

**Affiliations:** Department of Life Sciences, Imperial College LondonLondon, UK

**Keywords:** *Pseudomonas syringae pv. tomato*, proteomics, type three secretion systems, multiple reaction monitoring MS, proteomics shotgun, pathogenesis-related proteins

## Abstract

The plant pathogen *Pseudomonas syringae* pv.*tomato* (DC3000) causes virulence by delivering effector proteins into host plant cells through its type three secretion system (T3SS). In response to the plant environment DC3000 expresses hypersensitive response and pathogenicity genes (*hrp*). Pathogenesis depends on the ability of the pathogen to manipulate the plant metabolism and to inhibit plant immunity, which depends to a large degree on the plant's capacity to recognize both pathogen and microbial determinants (PAMP/MAMP-triggered immunity). We have developed and employed MS-based shotgun and targeted proteomics to (i) elucidate the extracellular and secretome composition of DC3000 and (ii) evaluate temporal features of the assembly of the T3SS and the secretion process together with its dependence of pH. The proteomic screen, under *hrp* inducing *in vitro* conditions, of extracellular and cytoplasmatic fractions indicated the segregated presence of not only T3SS implicated proteins such as HopP1, HrpK1, HrpA1 and AvrPto1, but also of proteins not usually associated with the T3SS or with pathogenicity. Using multiple reaction monitoring MS (MRM-MS) to quantify HrpA1 and AvrPto1, we found that HrpA1 is rapidly expressed, at a strict pH-dependent rate and is post-translationally processed extracellularly. These features appear to not interfere with rapid AvrPto1 expression and secretion but may suggest some temporal post-translational regulatory mechanism of the T3SS assembly. The high specificity and sensitivity of the MRM-MS approach should provide a powerful tool to measure secretion and translocation in infected tissues.

## Introduction

The Type III secretion system (T3SS) of *Pseudomonas syringae* transports helper and effector proteins, causing virulence. The plant innate immune system recognizes molecular microbial determinants, termed pathogen/microbe-associated molecular patterns (PAMPs/MAMPs), triggering a first line of the innate immune response (PAMP-triggered immunity, PTI). The second line of immune defense relies on the specific detection of injected effector proteins in the plant cytoplasm by resistance proteins, eliciting further immunity (termed effector-triggered immunity, ETI). PTI and ETI can combine to cause the hypersensitive response, a defense mechanism that largely defines the host specificity of *Pseudomonas syringae* pathovars (reviewed Zhang and Zhou, 2010). The effector repertoire of *Pseudomonas syringae* can counteract both PTI and ETI (reviewed Lindeberg et al., 2012).

*Pseudomonas syringae pv tomato* (DC3000) infects tomato (*Solanum lycopersicum*) and *Arabidopsis thaliana* and is the model to study T3SS-dependent plant pathogenesis (reviewed Xin and He, 2013). DC3000 genes coding for T3SS structural, helper and regulatory proteins are found in the *hrp/hrc* cluster that is flanked by effector loci, making up the Hrp tripartite pathogenicity island (Alfano et al., 2000). While specific biotic factors that trigger expression of the T3SS genes are poorly defined *in planta*, pH, temperature, the chemical composition of the plant apoplast, including nutrients, as well as physical interactions between host and pathogen stimulate expression of most genes within the pathogenicity island (Rico and Preston, 2008; Ortiz-Martin et al., 2010). The major transcription factors controlling transcription of genes involved in pathogenicity are the bacterial enhancer binding proteins HrpR and HrpS (Schumacher et al., 2006) and one of their regulatory targets, the extra-cytoplasmatic sigma factor HrpL (Rahme et al., 1992). HrpR and HrpS form an unusual, co-dependent enhancer binding protein pair in *Pseudomonas syringae* species that has been proposed to integrate various environmental cues (Jovanovic et al., 2011). However, how plant signals are perceived and transduced to allow for a coordinated expression and assembly of the T3SS and the secretion of helpers and effectors remains poorly understood.

Much of our knowledge about induction of the T3SS mediated pathogenicity and the delivery of effectors has been gained through genetics and their pathogenic phenotypes and translocation studies of effectors in bacterial cultures grown in various T3SS inducing media (e.g., van Dijk et al., 1999). Their roles in pathogenicity have been elucidated extensively by studying bacterial gene deletions (KOs) and by ectopically expressing these effector genes or combinations thereof in plant cells (Cunnac et al., 2011). However, our systems level understanding of plant-microbe interactions *in planta* is limited.

Recent advances in MS based proteomics now provides a powerful tool to begin to identify and quantify proteins from complex biological samples and could therefore provide novel proteomic level insights into the intricate biology during microbial plant infection. Both targeted MRM-MS and untargeted “shotgun” approaches can be applied to the same single proteome sample so that results from either approach are directly complementary. Using a hybrid Triple Quadrupole Ion trap instrument enables both these analyses to be carried out on the same instrument. Typically in gel free proteomics, total soluble proteins are extracted from biological samples and trypsin digested, producing a highly complex peptide mix which is analyzed directly by liquid chromatography coupled to MS analysis.

MRM-MS is performed with the MS in Triple Quadrupole mode, where a proteotypic signature peptide is preselected in the first analyzer (Q1), fragmented in the second quadrupole (Q2) and one or several fragments specifically measured in Q3. The detection of a particular peptide/fragment ion pair (termed transition) over time produces a quantifiable chromatographic peak. The pre-selection of peptides in Q1 and measurement of corresponding derivative fragments in Q3 allow for increased selectivity. A MRM-MS approach commonly requires prior information on the specific peptide(s) behavior during chromatography and MS behavior, so that analysis can be targeted to several peptides for each protein of interest, further increasing selectivity. Other advantages of this approach are high sensitivity, high reproducibility and low noise for the simultaneous measurement of up to (1000) transitions making it ideally suited for quantitative analysis.

Shotgun proteomics is a “bottom up” approach using the MS in Trap mode, here a survey scan is used to select for the most abundant peptides which are then analyzed by Enhanced Product Ion scans. The resultant peptide fragment ion spectra together with the cognate precursor peptide masses allow the identification of proteins from which they derived using protein database searches.

For a detailed review on both approaches, we refer to Maiolica et al. (2012). For absolute protein quantifications using MRM-MS, samples can be “spiked” with isotopically labeled protein standards of known concentration, such that transition peak areas derived from the sample (analyte) and the internal standard (IS) can be distinguished and the ratios between them used to calculate the concentration of the targeted protein in the sample. Commonly, signals from two protein- specific peptides and several transitions thereof can be combined in order to increase the precision of the quantitative analysis. A one to one ratio of two or more peptides from one protein is an indication for specificity of the selected transitions for the targeted protein.

We (i) performed both shotgun and MRM-MS to analyze the extracellular proteins of DC3000 after growth in *hrp* inducing media *ex planta*, (ii) and in relation to intracellular proteins and (iii) provide insights into the secretome composition and the temporal assembly of the T3SS and secretion process.

## Materials and methods

### Deletion of *hrpL* in DC3000

A gene deletion of *hrpL* was produced from *Pseudomonas syringae pv tomato* (DC3000) adapting a protocol for *Pseudomonas syringae pv phaseolicola* (1448a) (Zumaquero et al., 2010). Briefly, two ~700 bp sequences, corresponding to the 5′ and 3′ genomic flanking regions of *hrpL* were PCR amplified using primer pairs and the two PCR products fused by single-overlap extension PCR (Wurch et al., 1998), utilizing primer common homologous overlaps of 21 bp. Extension products were inserted into the pGEM-T vector (Promega). The BamHI fragment of the pGEM-T-nptII-BamHI plasmid, containing the FRT-flanked kanamycin resistance gene (*nptII*), was inserted into the pGEM-T derivative at a BamHI restriction site located within the 21 bp overlap sequence to yield the pKO*hrpL* suicide plasmid suitable for allele exchange in DC3000. Transformation with pKO*hrpL* was achieved utilizing an electroporation protocol described previously for *P. aeruginosa* (Choi et al., 2006). Kanamycin-resistant clones were screened for ampicillin sensitivity on LB-ampicillin plates to distinguish allelic exchange (a double recombination event) from whole plasmid integration (single recombination). Subsequently, a series of diagnostic PCR reactions combining *hrpL*-specific and *nptII*-specific primers were performed on genomic DNA in order to confirm *nptII* insertion at the correct chromosomal position (data not shown). The *nptII* resistance marker, flanked by Flippase Recognition Targets (FRT), was excised from the Δ*hrpL* (*nptII+*) locus via expression of the Flippase recombination enzyme (FLP) from the pFLP2 plasmid (Hoang et al., 1998). pFLP2 was removed via sacB-mediated counter-selection. Finally, the Δ*hrpL* locus was sequenced, showing the expected sequence that included the Flippase scar sequence. Where required, antibiotics were used at concentrations of: ampicillin 50 μg/mL; kanamycin (Km) 50 μg/mL; rifampicin 80 μg/mL.

### Strains and growth conditions

DC3000 and DC3000 Δ*hrpL* strains were routinely grown in King's broth (KB) medium at 28°C. For proteomics experiments, 250 ml pre-cultures of DC3000 were grown overnight in KB at 25°C, centrifuged (10 min, 4000 × g, 4°C), washed twice in 10 mM MgCl_2_ and re-suspended in HIM media [50 mM sodium phosphate buffer; 1.7 mM MgCl_2_; 1.7 mM NaCl; 7.6 mM (NH_4_)SO_4_] of different pH (as indicated), supplemented with 0.4% fructose as carbon source (HIM hereafter) (van Dijk et al., 1999). The re-suspended cultures were diluted in the same HIM media to obtain an optical density at 600 nm of 0.15 and grown at 25°C. Fifty milliliter samples were extracted for proteomic analysis at various time points (as indicated).

### Purification of doubly labeled protein standards

Protein standards for PSAQ were doubly labeled at arginine (L-Arginine- ^13^C_6_, ^15^N_4_, Sigma-Aldrich) and lysine (L-Lysine-^13^C_6_, ^15^N_2_, Sigma-Aldrich) residues *in vivo.* The coding sequences of *avrpto1* and *hrpA1* were PCR amplified and cloned into pET28b+ (Invitrogen) for overexpression of N-terminally histidine tagged proteins. An *Escherichia coli* Δ*argA* Δ*lysA* BL21 strain (Matic et al., 2011) was transformed with the resulting pET28b-*avrpto1* and pET28b-*hrpA1* constructs for highly efficient *in vivo* labeling and expression. Overnight cultures were grown in Gutnick minimal media (Schumacher et al., 2013) with 0.4% glucose and 10 mM NH_4_Cl, at 37°C, comprising labeled arginine and lysine and eighteen unlabeled amino acids at 1 mM concentrations each. Overnight cultures were diluted 50 fold for 100 ml day cultures grown in same media. When cultures reached an OD_600_ of 0.5, Isopropyl-β-D- thiogalactopyranosid (IPTG) was added to a final 1 mM and cultures grown for further 4 h. Cells were harvested by centrifugation at 4°C, 4000 rpm, for 20 min and pellets were stored at -20°C. Cell pellets were re-suspended in 10 ml 1x Gutnick medium with 7 M urea and cells disrupted by sonication for a discontinuous 7.5 min using a VWR7000 sonicator with 40% pulse, followed by centrifugation at 15,000 × g, 45 min at 4°C. Histidine tagged proteins were purified from the soluble fraction by nickel affinity chromatography on an Äkta FPLC system (GE Healthcare). The soluble fractions were loaded onto 1 ml HisTrap HP columns (GE Healthcare) and the column washed extensively with binding buffer (20 mM sodium phosphate, 0.5 M NaCl, pH 7.4, 7 M urea). Column bound proteins were eluted during a 20 ml imidazole gradient (from 0 to 200 mM) at 1 ml/min flow rate in an otherwise identical buffer as the binding buffer. Fractions containing the desired proteins, as judged by SDS-PAGE, were pooled and dialyzed against 1x Gutnick, 7M urea, 50% glycerol, 1 mM TCEP (Tris(2-carboxyethyl)phosphine), for storage at −80°C and to prevent oxidation. Protein concentrations of purified proteins were estimated using the Bradford assay based Bio-Rad protein assay and purities quantified using fluorescence band intensities of SYPRO^®^ Ruby stained SDS-Page (Supplementary Figure 1). For the quantitative analysis, specific protein standard concentrations of HrpA1 and AvrPto1were calculated, correcting for impurities.

### Proteome sample preparation

#### Intracellular proteins

Twenty-five milliliter of bacterial cultures were centrifuged and the resulting pellets and supernatants used for determining intracellular and extracellular (see below) MS analysis. Bacterial pellets were re-suspended in 1ml, 1x Gutnick (33.8 mM KH_2_PO_4_, 77.5 mM K_2_HPO_4_, 5.74 mM K_2_SO_4_, 0.41 mM MgSO_4_), 7 M urea solution and sonicated using a VWR7000 sonicator (2 s pulse and 2 s pause) at 40% amplitude for 2 × 7.5 min. Samples were centrifuged at 15,000 g at 4°C for 45 min to separate urea solubilized cell proteins from urea insoluble material. To 25 μl of the soluble fraction, the following were added: 10 μl of a standard protein mix of 0.51 μM HrpA1 and 5.48 μM AvrPto1 concentrations, 2.8 μl of 0.1 M TCEP, 245 μl of 50 mM NH_4_CO_3_ (freshly prepared) and 10 μl of xmg/ml sequencing grade trypsin from resuspending lyophilized modified trypsin as instructed by the manufacturer (Promega). Trypsin digestion was carried out for 3 h at 37°C and overnight at room temperature.

#### Extracellular proteins

Twenty-five milliliter of supernatants from above cultures were concentrated and digested using a filter aided sample concentration preparation protocol as described (Wisniewski et al., 2009). First, 4 μl of a protein standard mix (10.2 μM HrpA1 and 34.61 μM AvrPto1) were added to supernatants and the mixture concentrated to 200 μl and in same vial, the following were added: 1.8 ml of trypsin digestion buffer (100 mM Tris-HCl, pH 8, 50 mM NH_4_HCO_3_); and 10 μl of resuspended trypsin, as instructed by the manufacturer (Promega). Trypsin digestion was carried out for 5 h at 37°C and overnight at room temperature. Digested peptides were recovered by centrifugation at 5000 g at 4°C for 55 min, the supernatant was then dried using a vacuum centrifuge and resuspended in 200 μl of digestion buffer (100 mM Tris-HCl, pH 8, 50 mM NH_4_HCO_3_). Note that the addition of protein standards prior to concentration and trypsin digestion of proteins is predicted to internally control for potential losses during work up or incomplete trypsin digestion of the analyte proteins.

#### Mass spectrometry (MS)

Trypsin digested samples were analyzed on an ABSIEX 6500QTrap MS coupled to an Eksigent nano 400 LC system. The Eksigent LC system was configured to deliver capillary flow rates using the high flow (5–50 ul/min) module. The LC system was interfaced to the MS with an Ion Drive Turbo V source. Peptides were separated on an Agilent capillary column (Agilent ZORBAX 300 SB-C18 5 um 0.5 mm × 150 mm) using a gradient system of water (A) and acetonitrile (B) containing 0.1% formic acid. A short gradient of 0% B to 40% B over 15 min at a flow rate of 20 ul/min was used for the analysis The column was then washed with 90% (B) for 3 min before being returned to the initial conditions. Injection volumes were typically 5 ul. The column oven was heated to 40°C.

The MS, configured with low mass enabled, was used in “Trap” mode to acquire Enhanced Product Ion (EPI) scans for peptide sequencing and “Triple Quadruple” mode for Multiple Reaction Monitoring (MRM). In EPI scans, the protein identity based on detected peptides was evaluated using the Pro Group algorithm provided ABsiex software and total protein score (ProtScore) and percent coverage (% Cov) are given in Table 1. The total ProtScore expresses the total amount of evidence for a detected protein, while % Cov (95) gives the percentage of matching amino acids from identified peptides having confidence greater than or equal to 95 divided by the total number of amino acids in the sequence. For MRM analysis MS parameters were set to—Curtain Gas 35 psi, GS1 15 psi, GS2 30 psi, Interface heater on, TEM 125°C, DP 80, CXP 13, CE (collision energy) was set according to either empirically determined values or that estimated by MIDAS software (Applied Biosystems). For qualitative discover mode analysis, an IDA method consisting of a survey scan (300–100 m/z) followed by an Enhanced Resolution scan and then Enhanced Product Ion scans (230–1000 m/z) of the top 5 multiply charged ions in the survey scan was used. Dynamic background subtract was enabled and rolling collision energy was used at the default parameters. The MS source values were the same as used for the MRM analysis. MRM data analysis was performed using Analyst software (AB SCIEX), IDA data analysis was carried out using Protein Pilot 4.5 (ABSCIEX). Peptides were searched against a *P. syringae DC3000* database (Buell et al., 2003), which also contained common contaminants. Details on the peptides and transitions used for MRM-MS are listed in Supplementary Table 1. Protein concentrations were calculated by the relative peak intensities of the analyte (A) and the internal standard (IS) of known concentration.

**Table 1:**
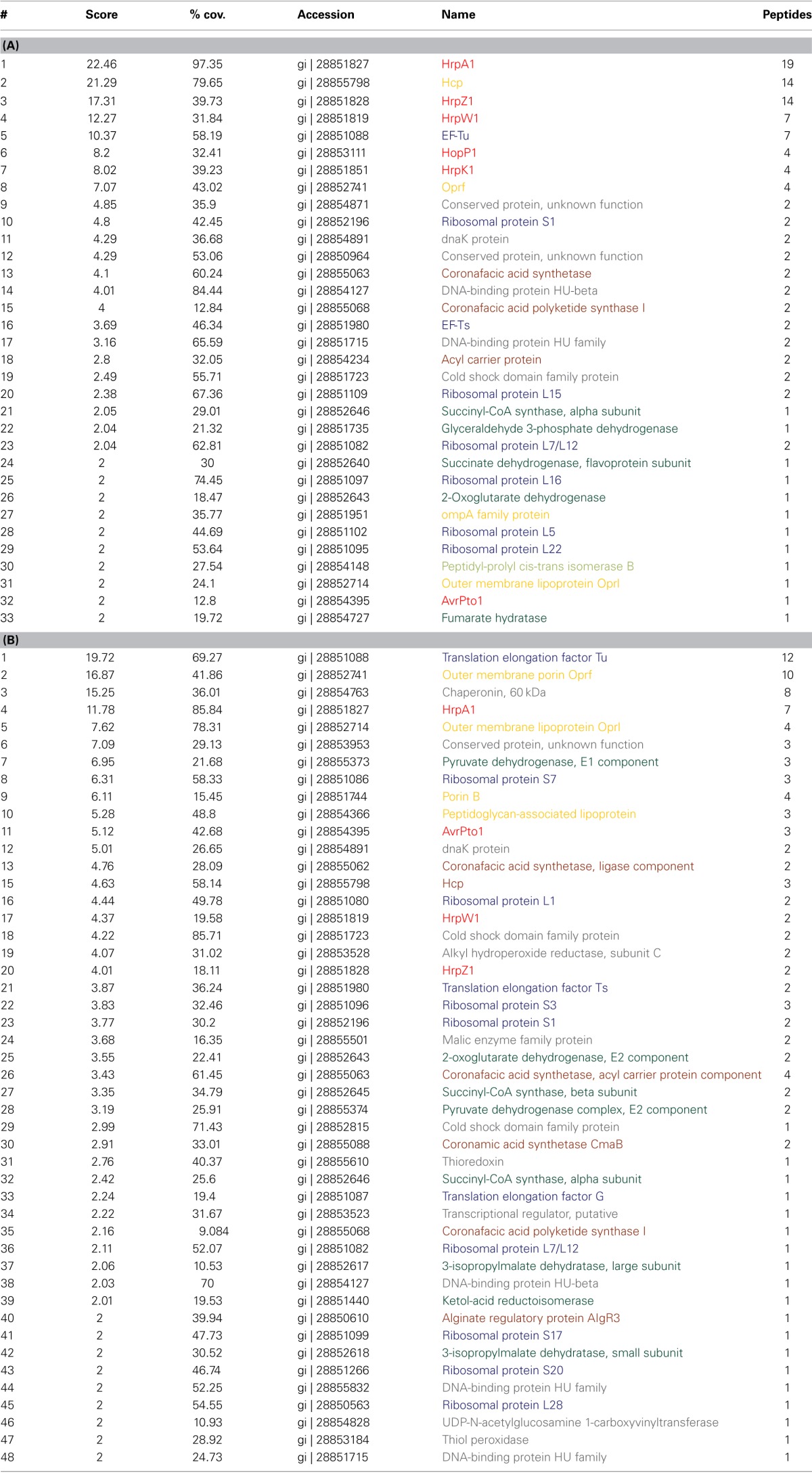
**Abundant proteins identified in the extracellular fraction (A) and cell fractions (B)**.

*Proteins are color coded: hrp/hrc gene products (red), pathogenicity associated (brown), extra-cytoplasmatic (yellow), translation machinery (blue), Krebs cycle and other enzymes (green), others (gray). The total protein scores (total ProtScore), % of sequence coverage and number of identified peptides per protein were as indicated*.

## Results

### Major intracellular and extracellular proteins

*hrp*-inducing medium (HIM) is commonly used to induce T3SS expression and effector secretion (Huynh et al., 1989) and we used HIM/fructose media for *hrp/hrc* induction. To gain initial insights into the intra and extra-cellular proteomic composition of DC3000, we carried out a shotgun MS approach (Maiolica et al., 2012) to identify proteins that are likely to be highly abundant in the cellular and extracellular fractions (Table 1). While detection of peptides depends both on their abundance and specific behavior in the MS, it is reasonable to assume that the peptides detected and their corresponding parent proteins are abundant, even though other proteins of high abundance may not be identified. Therefore, Table 1 does not represent a comprehensive list of intracellular and extracellular proteins. Fractions were derived from fractionating cell pellet (cellular hereafter) and supernatant (extracellular hereafter) by centrifugation from samples grown for 48 h in inducing media at pH 6.5. These conditions were sufficient for HrpA1 and AvrPto1 expression and secretion (further explored below). In order that extracellular and cell protein abundances were readily comparable, the extracellular fraction was concentrated by filtration to obtain the same final volume as the cell pellet derived proteomic sample. We tested isotopically- labeled AvrPto1 recovery enrichment using multiple reaction monitoring mass spectrometry (MRM-MS) before and after enrichment, showing that >80% of this standard was reproducibly retained using the extracellular fraction enrichment procedure (data not shown).

The most abundant apparent proteins in the extracellular fraction were those directly associated with T3SS mediated pathogenicity, HrpA1, HrpZ1, HrpW1, HopP1, and HrpK1 as well as hcp2 and the translation elongation factor Tu (EF-Tu) (Table 1A). Hcp2, the hemolysin-coregulated protein, is assumed to be an extracellular component of the Type VI secretion system (T6SS) (Sarris et al., 2010). The cellular fraction shows fewer T3SS associated proteins and of lesser relative abundance (Table 1B), indicating that HrpA1, HrpZ1, HrpW1, HopP1, and HrpK1 are largely secreted from DC3000 after induction with HIM. We note, however, that we mainly identified the T3SS structural protein HrpA1 as well as helper proteins HrpZ1, HrpW1, HopP1, and HrpK1 and only one effector protein AvrPto1 in the extracellular fraction. Detection of EF-Tu supports the findings that EF-Tu provides a major PAMP determinant during PTI (see Discussion). Other proteins identified in the extracellular fraction that may be associated with pathogenicity were: the acyl carrier protein, suggested to be involved in the regulation of virulence factors via N-acyl homoserine lactone in *P. syringae pv tabaci* (Taguchi et al., 2006); the coronafacic acid synthetase and coronafacic acid polyketide synthase, which are required for the synthesis of coronafacic acid. Coronafacic and coronamic acids together form the phytotoxin coronatine which is involved in growth and persistence in plant tissue and has also been shown to interfere with JA-mediated signaling in both *A. thaliana* and tomato (Uppalapati et al., 2007).

The predominant proteins in the supernatant fraction that have not been linked to pathogenicity are mainly ribosomal proteins and metabolic enzymes of the Krebs cycle, i.e., proteins that are expected to exist in large numbers in the cell. Furthermore, several outer membrane proteins, the chaperone DnaK and a few conserved proteins were detected. Of the 48 cellular proteins, 11 are involved in translation and 9 are major metabolic enzymes. Nineteen other proteins were also found in the supernatant fraction. Of these, the cellular alkyl hydroperoxide reductase, thioredoxin, and thiol peroxidase may play a role in alleviating oxidative stress brought about by reactive oxygen species (ROS) as a component of plant defense (e.g., Somprasong et al., 2012). Overall, these data indicate discrete secreted and cellular protein sets in the pellet and supernatant fractions and, excluding the possibility of major cell lysis accounting for presence of proteins in the supernatant fraction, implying that many of the proteins identified are indeed secreted.

### MRM-MS selectivity and sensitivity for secreted AvrPto1 and HrpA1

To gain more quantitative insights into T3SS expression and assembly and the secretion process of DC3000, we subsequently determined the cell and extracellular concentrations of HrpA1 and AvrPto1 by MRM-MS, following induction with HIM-fructose growth media over time and at variable pH. MRM-MS is used increasingly in quantitative systems biology but has hitherto been mainly applied to quantify the abundance of proteins in cells (Maiolica et al., 2012). We reasoned that advances in MRM-MS approaches would offer a number of advantages compared to previous *Pseudomonas syringae* secretome studies, predominantly utilizing Western blotting (Hirano et al., 1999; Collmer et al., 2000). In particular increased accuracy, sensitivity and selectivity and the potential for higher throughput using multiplexing capacities. Isotopically labeled HrpA1 and AvrPto1 were utilized in order to perform protein standard absolute quantification (PSAQ). PSAQ offers a number of advantages compared to other internal standard strategies, such as AQUA or Qconcat, due to the biochemical identity of the labeled full-length protein with that of the analyte protein. Both protein species are subject to identical sampling handling such that any decrease in output signal, for instance as a result of incomplete trypsin digestion, is common to both and thus controlled for internally (Picard et al., 2012). Purities of labeled HrpA1 and AvrPto1 were determined to be 96 and 52% (Supplementary Figure 1) by relative band fluorescence intensities of SYPRO^®^ Ruby stained SDS-PAGE gels. The *in vivo* labeling efficiencies for HrpA1 and AvrPto1 were 100 and 99.7%, respectively, as determined by MS. Given the large numbers of proteins and protease derived peptides thereof in proteomic approaches, detection of a specific protein remains challenging in “gel-free” proteomic approaches.

**Figure 1:**
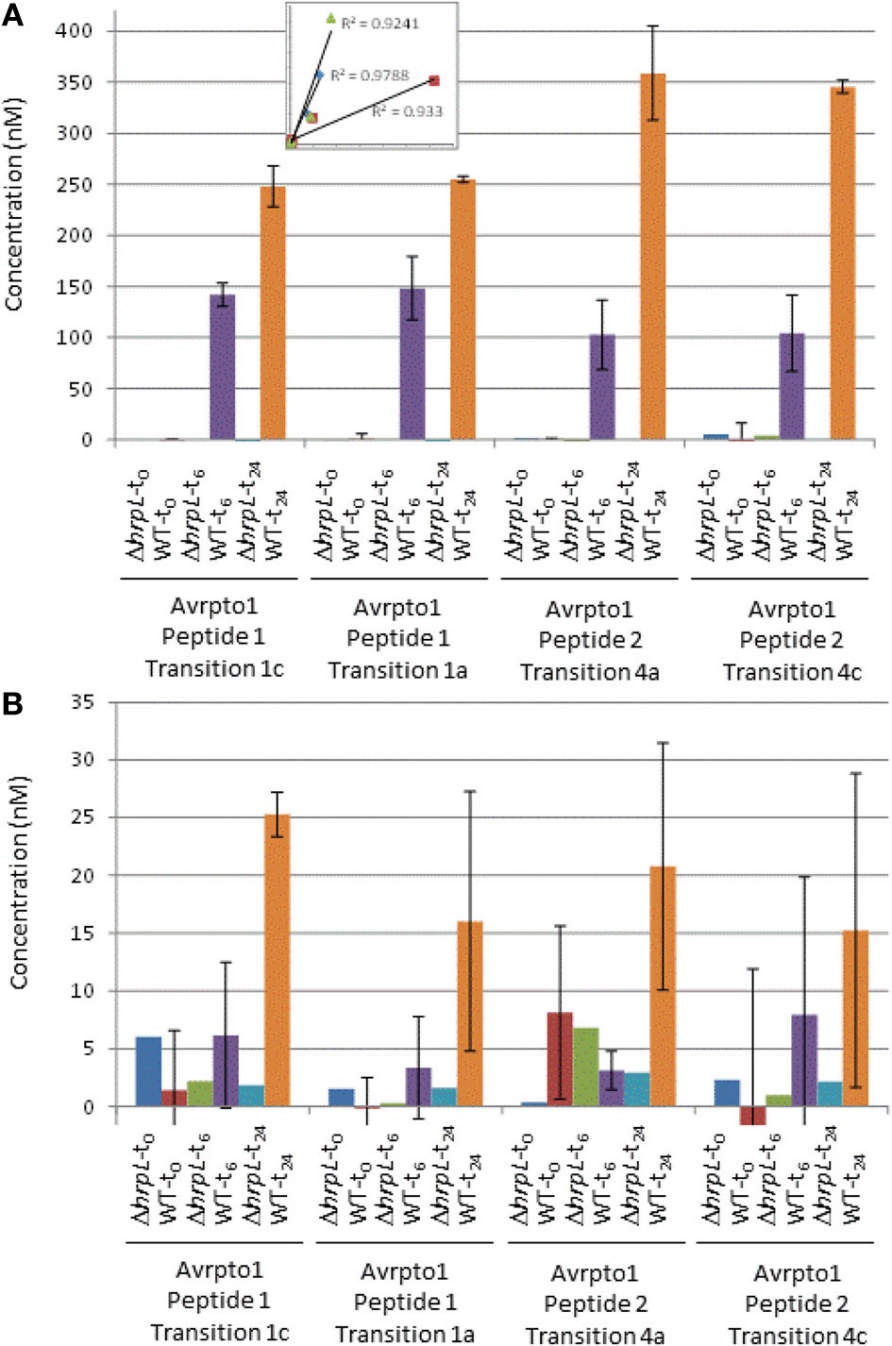
**Absolute quantification of AvrPto1**. Main: Absolute protein concentrations in nM in the extracellular fractions **(A)** and cell fractions **(B)** from DC3000 and DC3000D*hrpL* after 0, 6, and 24 h after induction with HIM /fructose media (pH 6.5), based on independent calculations from four transitions (as indicated). Error bars indicate one standard deviation from the mean from two biological replicates. Inset in Figure 1A shows the correlation of extracellular fraction sample peak intensities between AvrPto1 transitions and peptides (see text).

To confirm selectivity of the chosen transitions (Table), we produced an in frame deletion of *hrpL* in DC3000 (DC3000Δ*hrpL*), the transcriptional activator of *hrpA1* and *avrpto1*. As expected, only background levels were observed for transitions (Table 1) of AvrPto1 and HrpA1 in samples of DC3000Δ*hrpL* grown in inducing media (Figure 1), indicating that selected transitions are specific to *hrpL*-dependent expression of *avrpto1* and *hrpA1*. To investigate the accuracy and sensitivity of our approach we compared MRM-MS with PSAQ stoichiometries of four transitions derived from two different AvrPto1 peptides (Table 1). The good correlations between two transitions from both extracellular fraction derived peptides and the correlation of averaged transitions between those peptides (Figure 1A inset) suggest good technical reproducibility. Figure 1 shows the determined MS-sample AvrPto1 concentrations based on four individual transitions in the pellet and extracellular fractions (Materials and Methods). We found good reproducibility between two biological replicates in quantifying AvrPto1 in excess of 100 nM concentrations in the MS sample, supporting our approach. When *AvrPto1* transitions were averaged, we also detected AvrPto1 above background levels in the pellet fraction after 48 h of induction, at a concentration of 19.3 nM (± 4.7 standard deviation), indicating a lower limit of detection. Taken together, these results indicate MRM-MS allows for selective and sensitive quantification of AvrPto1 in complex samples.

**Figure 2:**
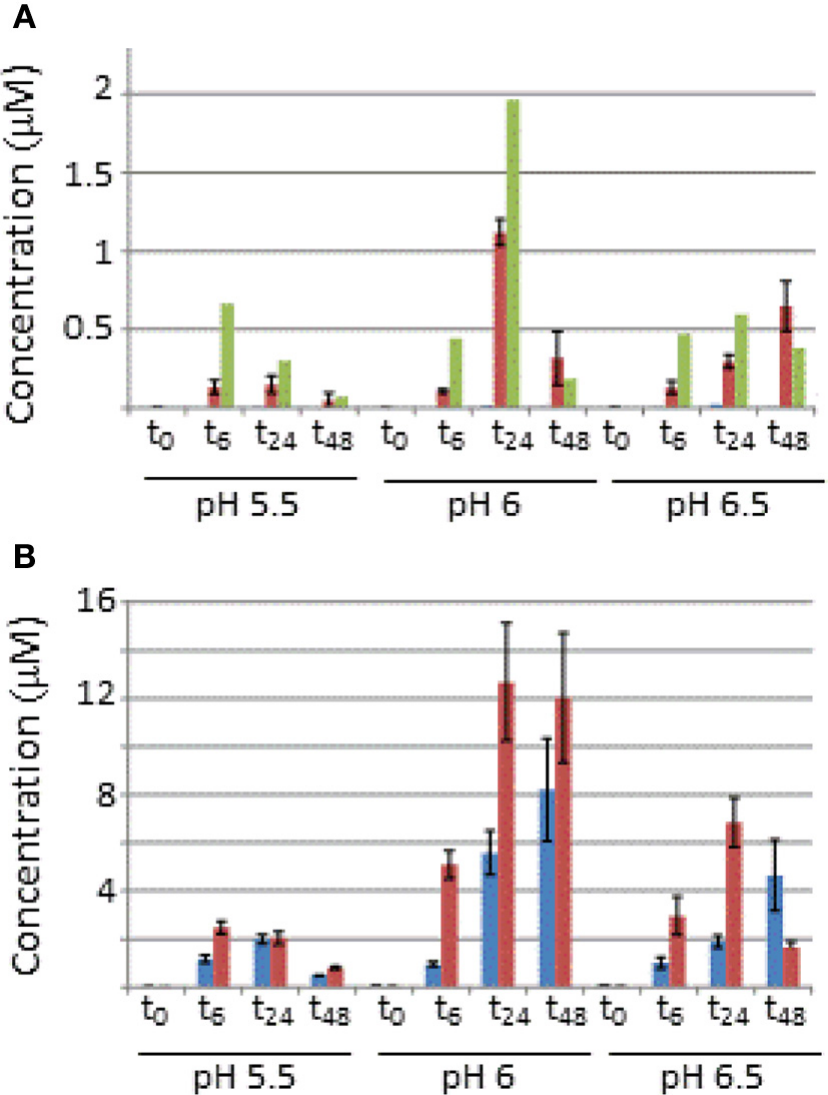
**Absolute concentrations of AvrPto1 (A) and HrpA1 (B) in the pellet and extracellular fractions after 0, 6, 14, and 48 (*t*_0_–*t*_48_) after induction in HIM-fructose media at different pH (as indicated)**. Concentrations from cell (blue) and extracellular fractions (red) are in μM and in the case for AvrPto1, concentrations were also normalized against OD_600_ (green bars) of the cultures. Judged by OD_600_ growth was very similar between cultures, reaching OD_600_ values of 1.7, except for *t*_48_ in the case of pH 5.5, which only grew up to 0.64. Error bars represent one standard deviation from the mean from the four averaged transitions of AvrPto1 and the two available transitions of HrpA1 (see text).

The absolute quantification using internal standards for HrpA1 was complicated by the fact that identifiable peptide 1 of HrpA1 was N-terminal. Because the HrpA1 internal standard was histidine tagged, we could not compare signal intensities between the N-terminal peptide from samples with that of the standard. We therefore based our absolute quantification of HrpA1 on the internal peptide alone and could therefore only average protein concentrations based on the two transitions from that peptide, explaining the lower accuracy in determining HrpA1 concentrations compared with those for AvrPto1 (Figure 2).

### AvrPto1 and HrpA1 expression are pH dependent and AvrPto1 is readily secreted

Figure 2 shows the concentration of AvrPto1 and HrpA1 in the cellular and extracellular fractions, determined by MRM-MS, of samples grown at different pH after 0, 6, 24, and 48 h. Recall that sample volumes and calculations were adjusted so that protein amounts in both fractions are comparable in absolute terms. AvrPto1 is almost exclusively found in the extracellular fraction at concentrations in the low micro-molar range, indicating that expressed AvrPto1 is readily secreted under all conditions. AvrPto1 is most abundant when grown at pH6, this holds true when correcting for cell density (green bars in Figure 2A, see Supplementary Figure 2 for growth curves). HrpA1 is about 10 times more abundant than AvrPto1, consistent with its role as the primary T3SS structural protein. Also, we found HrpA1 to be associated with both fractions, suggesting that a proportion of the T3SS pili disconnect from the cellular base and/or that HrpA1 fails to fully assemble into the pilus structure at its tip, both a potential result of mechanical strain during culture shaking or centrifugation. Comparing total AvrPto1 and HrpA1 amounts, we found no evidence to suggest that either protein is expressed earlier than the other, although the timeframe of our analysis may lack the necessary resolution. After 48 h of growth, when cells grown at pH 6 and 6.5 reached stationary phase (Supplementary Figure 2), AvrPto1 and HrpA1 levels differed significantly from their previous levels at 24 h, suggesting that these proteins may be degraded under starvation.

### HrpA1 undergoes rapid extracellular N terminal modification

**Figure 3:**
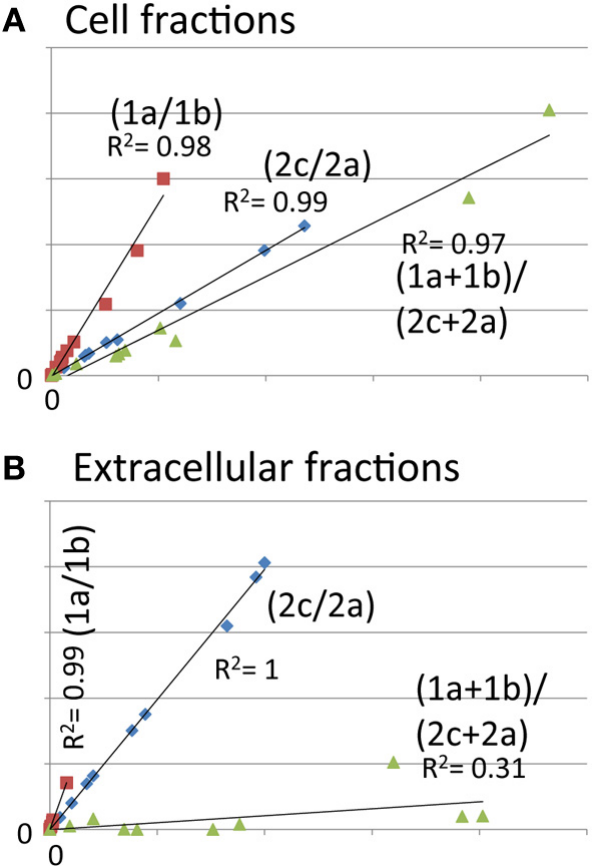
**Abundance of the N-terminal peptide of HrpA1 is very low in the extracellular fractions, indicating low abundance of full length HrpA1**. Scatter plots of analyte peak counts of HrpA1 from twelve cell **(A)** and extracellular **(B)** samples (as in Figure 2). **(A)** Correlation of peak counts for transitions 1a and 1b from peptide ISATATNAK (red squares), 2c and 2a for peptide LTNLGNSAVGGVGGALQGVNTVASNATLQK (blue diamonds), and the sums of transitions from both peptides (green triangles). R squared values for trend lines are as indicated.

**Figure 4:**
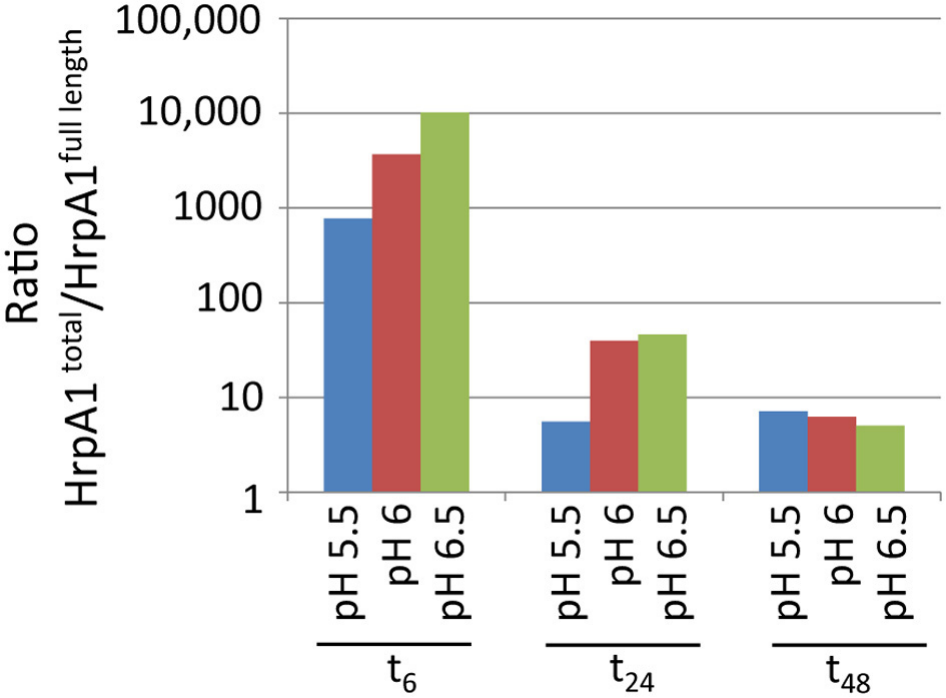
**Relative abundances over time of peptides ISATATNAK (indicating total HrpA1) and LTNLGNSAVGGVGGALQGVNTVASNATLQK (indicating full length HrpA1) in the extracellular fractions (in semi log)**. Abundance ratios were determined by dividing the sum of transition pair signals of the former peptide by the sum for the latter (in arbitrary units). The equivalent ratios across 12 cell bound samples was constant at 1.62 (± 0.09 SE).

When correlating signals from HrpA1 transitions and peptides from 24 cell and extracellular fractions we found excellent correlations between the transition pairs for both peptides. However, when correlating the sums of transitions from one peptide with the other, we did not find a good correlation, as we did for AvrPto1 (Figure 1A inset), initially suggesting that the selectivity of the MRM-MS for HrpA1 peptides was poor. However, when comparing transition pairs and their sums of the cell and extracellular fractions separately, we found very good correlations between the abundances of both HrpA1 derived peptides in the cell fractions (Figure 3A), but not in the extracellular fraction (Figure 3B). While stoichiometry between HrpA1 peptides ISATATNAK (HrpApep1) and LTNLGNSAVGGVG GALQGVNTVASNATLQK (HrpApep2) was constant in all cell fractions, we found a drastic under-representation of the latter, N-terminal peptide in the extracellular fraction. This suggested that HrpA1 is post-translationally modified at the N-terminal upon secretion or thereafter. Roine and colleagues reported the purification of HrpA1 from cell supernatants of *Ps* DC3000 grown in HIM inducing media and found purified HrpA1 proteins to be N-terminally truncated after amino acid positions 16, 35, or 41 (Roine et al., 1997). Because our targeted MRM-MS approach relies on the integrity of signature peptides, the low abundance of the HrpA1 in the extracellular fraction here is fully consistent with these findings, demonstrating a high selectivity of both detected HrpA1 peptides—where present—by MRM-MS. Our results directly show that HrpA1 is exclusively truncated at or after secretion and not intracellularly. We have previously shown that outer membrane porins of *E. coli* are associated with the cell bound fractions, using the same protocol to separate cells from supernatant (Schumacher et al., 2013). We therefore propose that HrpA1 truncation does not occur in the periplasm. To gain quantitative insights into the extent of truncation and its kinetics, we calculated the ratios of MRM-MS signals for both HrpA1 peptides in the extracellular fraction (from same samples as before). We were not able to independently determine the absolute concentrations for the N-terminal peptide as our internal standard carried the N-terminal histidine tag. However, since the same signal ratio calculations for the cell fractions were constant 1.62 (± 0.09 SE), relative abundances and change could be determined (Figure 4). Surprisingly, we found that after 6 h induction, almost all secreted HrpA1 was truncated, whereas at later stages a larger proportion of full length HrpA1 was present in the extracellular fraction. Taking the 1.62 average ratio derived from the cell fractions, where HrpApep1 and HrpApep2 are stoichiometrically equal, these results suggest that after 48 h, 23–32% of HrpA1 is in full length form in the extracellular fraction (see Discussion).

## Discussion

As judged by shotgun proteomics, the T3SS structural protein HrpA1 and helper proteins HrpZ1, HrpW1, HopP1, and HrpK1 are among the most abundant seven proteins (Table 1). HrpZ1, HrpW1, HopP1, and HrpK1 are involved in translocation of effector proteins, but are themselves not translocated (Kvitko et al., 2007). HrpZ1, HrpW1, and HopP1 are hairpin proteins that appear unique to and ubiquitous among phytopathogens employing the T3SS system for infection. HrpZ1 forms ion conductive pores and binds to the plant wall and lipid bilayers (Lee et al., 2001), but only elicits a hypersensitive response by itself at very high concentrations (Tampakaki and Panopoulos, 2000). HrpK1 is also involved in translocation and functions synergistically with HrpZ1, HrpW1, HrpK1, and HopP1 to elicit a hypersensitive response in host and non-host plants (Kvitko et al., 2007). The ensemble identification of highly abundant HrpA1, HrpZ1, HrpW1, HopP1, HrpK1, and AvrPto1 in the extracellular fraction suggests that their expression and secretion occurs at least partly independently from any specific plant determinants, yet could elicit a plant specific response. In good agreement, Haapalainen and colleagues also identified HrpA1, HrpZ1, HrpK1, HopP1, HrpW1, and HopAK1 and no other effector protein commonly associated with pathogenicity of DC3000 after growth in inducing media in the extracellular fraction when using 2D gel electrophoresis and TOF based MS. However, because in their work extracellular proteins were targeted due to their relative abundance in relation to non-inducing media, most of the proteins shown in Table 1, were not identified (Haapalainen et al., 2012). We identified a large number of proteins directly associated with translation (EF-Tu, EF-Ts, and ribosomal proteins), as well as enzymes of carbon metabolism, in the supernatant fraction. The very low abundance of full length HrpA1 in the extracellular fraction, despite its high abundance in cell fractions rules out the possibility that these proteins of intracellular function in the extracellular fraction were caused by significant cell disruption during sample growth or centrifugation, although we cannot determine the exact proportion of any protein that may have escaped the cytoplasm due to some cell lysis. The presence of particular abundant proteins in the extracellular fraction may however be of biological significance with regards to plant immunity. It is established that epitopes of EF-Tu elicit the plant innate immune response (Kunze et al., 2004 and reviewed in Newman et al., 2013), although its presence in the *Pseudomonas syringae* extracellular fraction has not, to our knowledge, been physically evidenced. The most likely communal feature of many of the proteins found in the extracellular fraction is high cellular abundance and we speculate that their detection in the extracellular fraction is simply a consequence of a few cells dying or being otherwise disrupted. Similarly, Krebs cycle enzymes are likely to be among the most abundant in the cell. While accurate measurements of cellular proteins are scarce, two proteomic studies suggested that the most abundant intracellular proteins in *E. coli* included EF-Tu, a ribosomal subunit RpsV, the Krebs cycle enzyme malate dehydrogenase and dnaK (Lu et al., 2007; Taniguchi et al., 2010). Since copy numbers of these proteins per cell are measured in tens of thousands, it seems feasible that a low level of cell lysis may account for their relatively abundant presence in the *Pseudomonas syringae* extracellular fraction. Importantly, this observation leads us to hypothesize that plants may have evolved to recognize epitopes of such abundant housekeeping proteins for PAMP-triggered immunity, as even a small number of cell lysis in infected tissue would release a large number of proteins that could suffice for detection by the plant. Furthermore, these proteins are usually vital and highly conserved, such that there exists strong negative selection against mutations that could otherwise enable evasion of plant detection. A systematic bio-informatics approach across bacterial species evaluating the relationship between known protein epitopes involved in PAMP triggered immunity and their conservation across species may substantiate this hypothesis. If supported, proteins in Table 1 may include not yet identified candidates involved in PTI.

The highest extracellular fraction concentrations of HrpA1 and AvrPto1 were found after 24 h when cells were grown in HIM-fructose media at pH 6. This is consistent with findings using AvrPto1 specific antibodies in Western blotting (Hirano et al., 1999). Our results suggest that this is a consequence of higher *avrpto1* and *hrpA1* expression as the total HrpA1 and *AvrPto1* amounts were also maximal under these conditions, supporting the idea that *Pseudomonas syringae* senses the chemical environment of the apoplast as important parameter for pathogenesis (Rico et al., 2011).

Protein concentrations determined in the MS samples (as in Figures 1, 2) can yield approximate protein copy numbers produced per cell over time. For instance, assuming an OD_600_ of 1 represents a density of 1.11 ^*^ 10^9^ cells/ml, as for *E. coli* [the similar sizes of *E. coli* (1 μm^3^, Schumacher et al., 2013) and *Pseudomonas syringae* (1.26 μm^3^, Monier and Lindow, 2003) may support this correlation], an average *Pseudomonas syringae* cell grown for 6 h in HIM-fructose at pH 6 would have produced ~450,000 HrpA1 and 8000 AvrPto1 per hour (assuming no degradation occurs). This suggests that the energetic and material cost of T3SS production is considerable, at least in our batch culture conditions. We also found that hourly expression between 6 and 24 h (corrected for OD_600_ and time) under these conditions is lower for HrpA1 (190,000) and about constant for AvrPto1 (12,000), suggesting that full induction of *hrpA1* and *avrpto1* expression occurs within the first 6 h in HIM-fructose media.

One surprising observation was that HrpA1 appears largely truncated in the extracellular fraction but not in the cell fractions, suggesting that an as-yet unidentified protease acts extra-cellularly on HrpA1 particularly during the first 6 h after induction (Figure 4). Roine and colleagues showed that HrpA1, purified from the extracellular fractions of DC3000 comprised a mixture of differentially N-terminal cleaved HrpA1 by Edman degradation (Roine et al., 1997). The first 41 amino acids of HrpA1 comprise three cleavage sites that are preceded by a threonine four positions N-terminal to the cleavage before asparagine (TXXXN) (Table 2 legend), suggesting that proteolysis is sequence specific. The different truncated versions retain the capacity to self-assemble into pili structures *in vitro*. Deletions and transposon insertion mutations studies in the N-terminal moiety of *hrpA1* of DC3000 did not reveal any obvious phenotype with regards to auto-agglutination, pathogenicity or the capacity to elicit the hypersensitive response (Taira et al., 1999). A protein sequence search of the N-terminal sequence of HrpA1 of DC3000 against microbial protein databases suggested that this sequence is specific to a subset of *Pseudomonas syringae* pathovars only (listed in Table 2) and the DC3000 related pathovars *tabaci* and *pisi* have only rudimentary similarities within this sequence and no discernible TXXXN motif. While a potential role or mechanism for HrpA1 truncation remains to be established some sort of temporal control over the extent of N terminal processing is evident.

**Table 2:** **Sequence conservation of HrpA1 N-terminal sequence among *Pseudomonas syringae* pathovars**.

***Pseudomonas syringae* pathovars**	**% Identity**	**% Positives**
Tomato (DC3000)	100	100
Delphinii	100	100
Actinidae	100	100
Theae	98	100
Maculicula	98	97
Amygdali	93	97
Avellanae	93	97
Tagetis	82	89
Oryzae	54	63
Coronafaciens	54	63
Savastanoi	53	66
Phaseolicola	53	66
Glycinae	53	66

Sequence identities and % positives were identified by BlastP search of the 41 N-terminal sequence of HrpA1 from Pseudomonas syringae pv tomato (DC3000: (^1^MVAFAGLTSKLTNLG^*^NSAVGGVGGALQGVNTVAS^*^NATLQK^41^^*^NIL, asterisks indicating cleavage sites) against the microbial protein database.

MS based proteomics approaches increasingly allow the simultaneous detection and semi-quantification of hundreds of proteins (shotgun approaches) as well as their accurate quantification and using MRM-MS (Picotti et al., 2013). This offers novel opportunities to study plant-microbe interactions both from a systems and molecular perspective. For instance our biologically unbiased shotgun approach identified many proteins as abundant in the extracellular fraction whereas more targeted approaches would have missed this complexity. MRM-MS analysis allowed the faithful quantification of AvrPto1 in the nano-molar range and it is expected that method optimization will further enhance the sensitivity of MRM-MS by a few orders of magnitude. Hitherto, much of our understanding of plant-microbe interactions is based on studies with either plant or microbe—as is this one—using models or conditions that only imperfectly reflect the *in vivo* situation. Ongoing rapid advances in MS based proteomics and in particular MRM-MS should in principle allow detection and quantification of plant and microbe derived proteins from infected tissues in the near rather than the distant future (Picotti and Aebersold, 2012), allowing a far more complete picture of the plant-microbe interaction to be drawn.

### Conflict of interest statement

The authors declare that the research was conducted in the absence of any commercial or financial relationships that could be construed as a potential conflict of interest.

## Supplementary material

Supplementary Table 1 **Peptides and transitions used for MRM-MS**. Shown are the charge/mass ratios of the precursor and product ions, chromatographic retention times, parent protein, the peptide sequences and transition IDs, specifying if derived from the analyte (a) or internal standard (IS) as well as the used collision energies in Q2. ^*^, not used as histidine tagged (see text).

Supplementary Figure 1 **Fluorescence image (A) and lane evaluation (B) of SYPRO ruby stained SDS Page of isotopic labeled HrpA1 and AvrPto1 protein standards**. Purities were estimated by band intensities of the expected SDS Page gel mobilities of 18329 Da (AvrPto1) and 11387 (HrpA1) relative to total protein intensities.

Supplementary Figure 2 **Growth curves of DC 3000 in HIM-fructose media at pH 5.5 (blue), 6 (red) and 6.5 (green), as judged by optical density at 600 nm (OD_600_)**. At 48 h, cells had entered early stationary phase as judged by slowing growth.
