# Supplementary figures and images for: Differential secretome analysis of Pseudomonas syringae pv tomato using gel-free MS proteomics
Source: Front Plant Sci. 2014 Jul 4;5:242. doi: 10.3389/fpls.2014.00242 (PMC4082315; doi:10.3389/fpls.2014.00242)

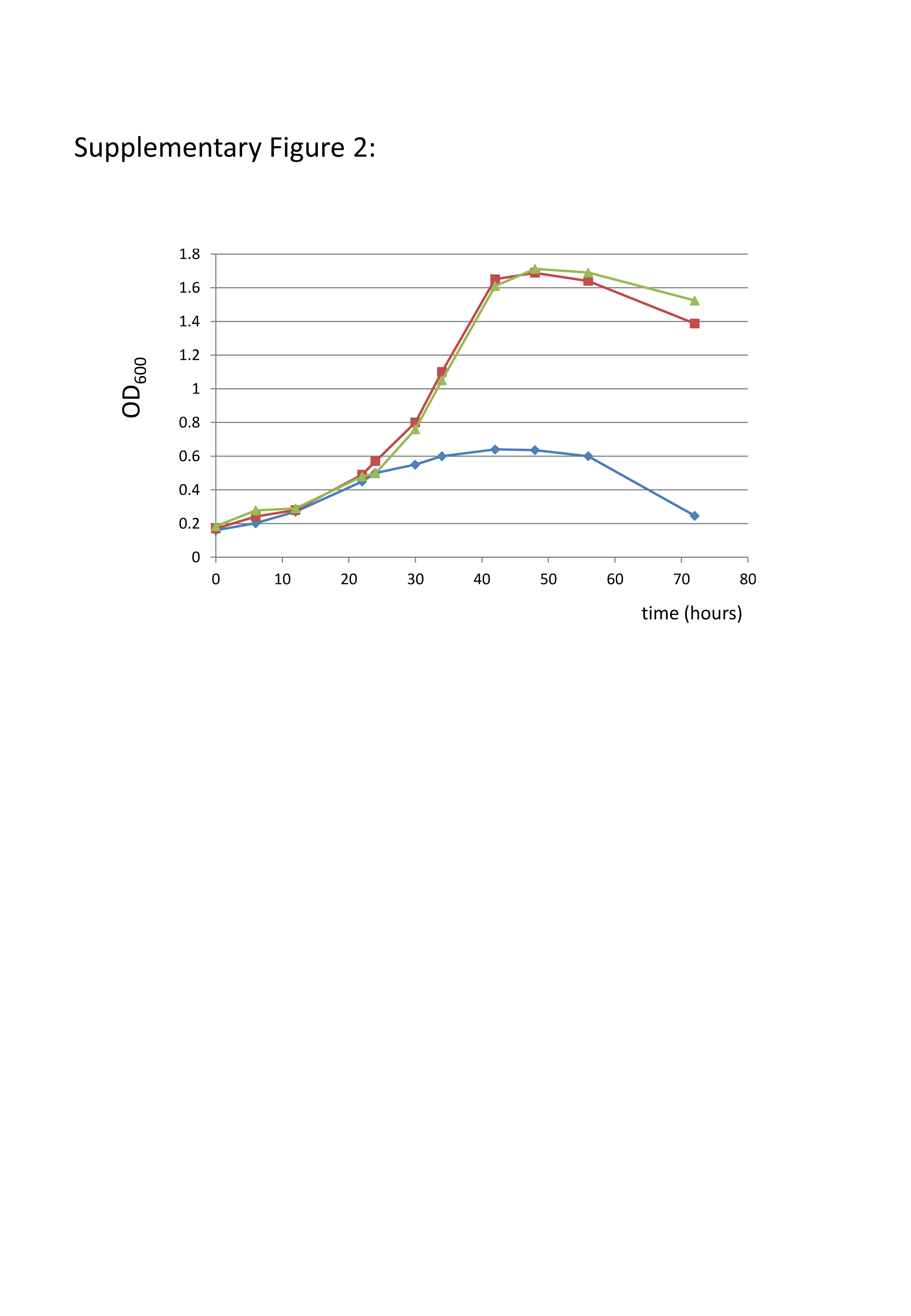

Supplement: Supplementary Table 1 — Peptides and transitions used for MRM-MS. Shown are the charge/mass ratios of the precursor and product ions, chromatographic retention times, parent protein, the peptide sequences and transition IDs, specifying if derived from the analyte (a) or internal standard (IS) as well as the used collision energies in Q2. *, not used as histidine tagged (see text). [file Presentation1.ZIP › 82666_Schumacher_Sup Fig 2.TIF]

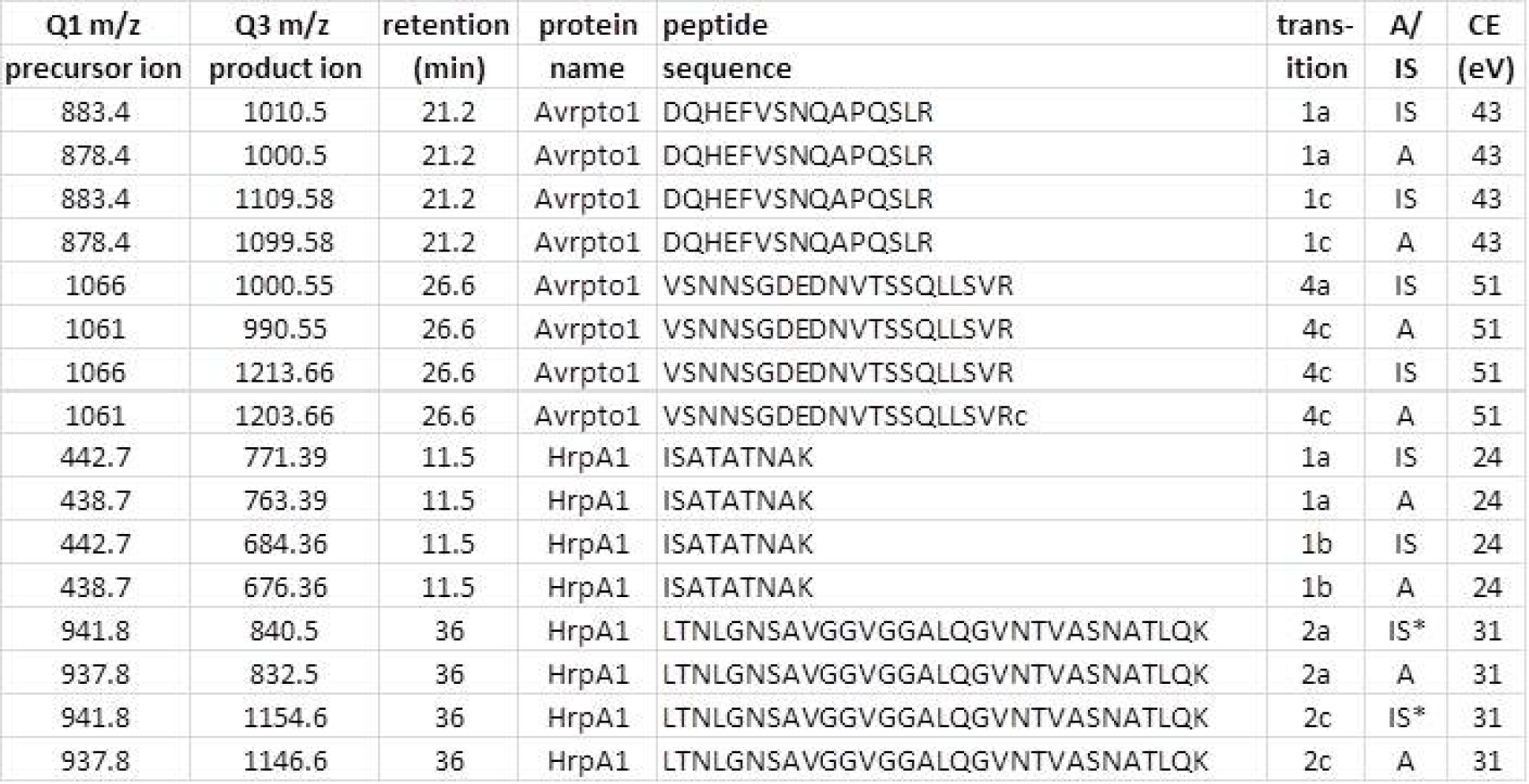

Supplement: Supplementary Table 1 — Peptides and transitions used for MRM-MS. Shown are the charge/mass ratios of the precursor and product ions, chromatographic retention times, parent protein, the peptide sequences and transition IDs, specifying if derived from the analyte (a) or internal standard (IS) as well as the used collision energies in Q2. *, not used as histidine tagged (see text). [file Presentation1.ZIP › 82666_Schumacher_Sup Tab 1.TIF]

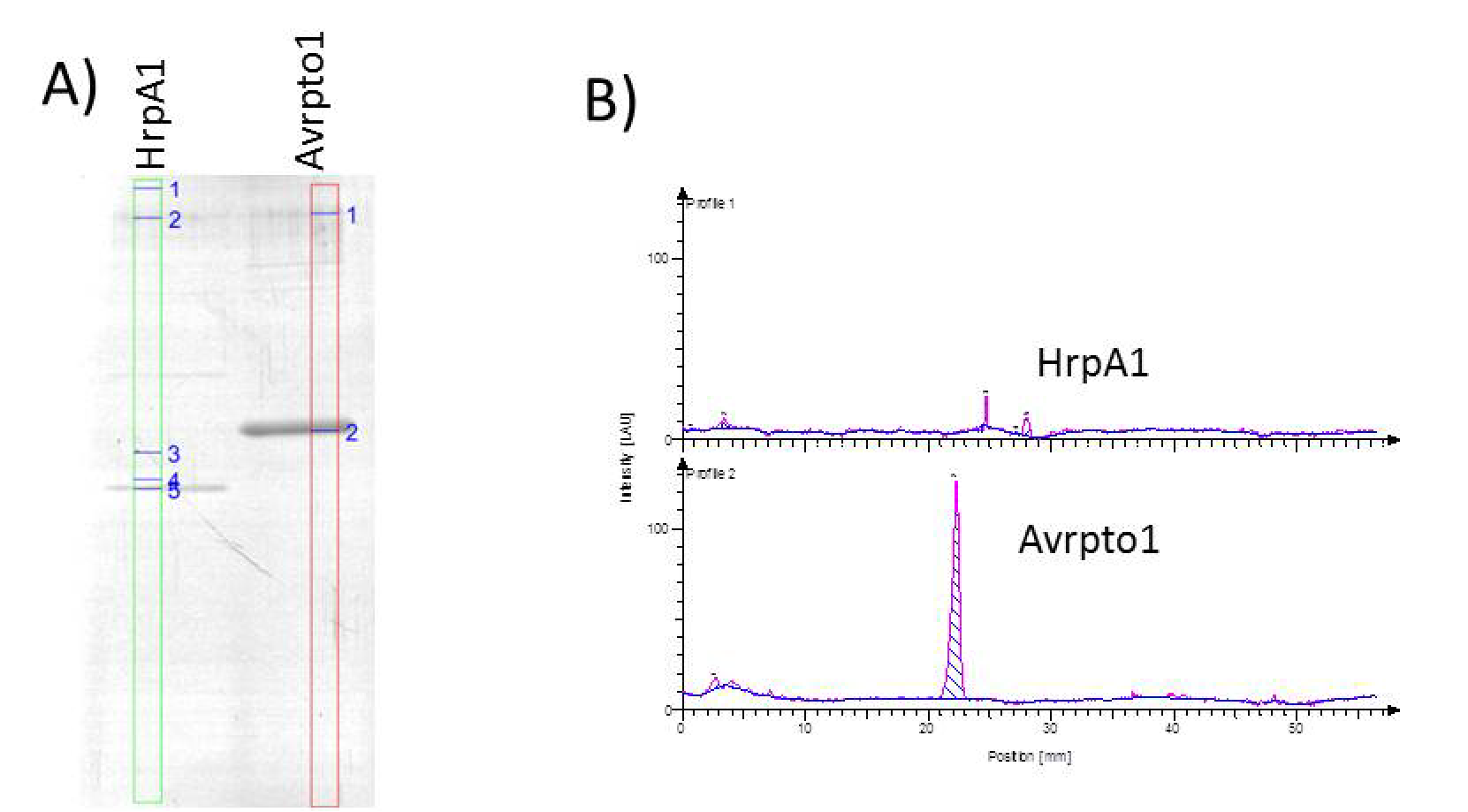

Supplement: Supplementary Table 1 — Peptides and transitions used for MRM-MS. Shown are the charge/mass ratios of the precursor and product ions, chromatographic retention times, parent protein, the peptide sequences and transition IDs, specifying if derived from the analyte (a) or internal standard (IS) as well as the used collision energies in Q2. *, not used as histidine tagged (see text). [file Presentation1.ZIP › 82666_Schumacher_Sup Fig 1.TIF]
